# Analysis of Signal Transduction Pathways Downstream M2 Receptor Activation: Effects on Schwann Cell Migration and Morphology

**DOI:** 10.3390/life12020211

**Published:** 2022-01-29

**Authors:** Elisabetta Botticelli, Michael Sebastian Salazar Intriago, Roberta Piovesana, Ada Maria Tata

**Affiliations:** 1 Department of Biology and Biotechnologies Charles Darwin, Sapienza University of Rome, 00185 Roma, Italy; botticellielisabetta9@gmail.com (E.B.); michaelsebastian.salazarintriago@uniroma1.it (M.S.S.I.); roberta.piovesana@umontreal.ca (R.P.); 2 Department of Neurosciences, Université de Montréal, Montréal, QC H3C 3J7, Canada; 3 Research Centre of Neurobiology Daniel Bovet, 00185 Rome, Italy

**Keywords:** Schwann cells, M2 muscarinic receptor, β1-arrestin, mTOR pathway, AMPK, cell migration

## Abstract

Background: Schwann cells (SCs) express cholinergic receptors, suggesting a role of cholinergic signaling in the control of SC proliferation, differentiation and/or myelination. Our previous studies largely demonstrated that the pharmacological activation of the M2 muscarinic receptor subtype caused an inhibition of cell proliferation and promoted the expression of pro-myelinating differentiation genes. In order to elucidate the molecular signaling activated downstream the M2 receptor activation, in the present study we investigated the signal transduction pathways activated by the M2 orthosteric agonist arecaidine propargyl ester (APE) in SCs. Methods: Using Western blot we analyzed some components of the noncanonical pathways involving β1-arrestin and PI3K/AKT/mTORC1 signaling. A wound healing assay was used to evaluate SC migration. Results: Our results demonstrated that M2 receptor activation negatively modulated the PI3K/Akt/mTORC1 axis, possibly through $\beta_{1}$-arrestin downregulation. The involvement of the mTORC1 complex was also supported by the decreased expression of its specific target $\mathrm{p-p}70\;S6K^{Thr389}$. Then, we also analyzed the expression of $\mathrm{p-}{\mathrm{AMPK}\alpha }^{thr172}$, a negative regulator of myelination that resulted in reduced levels after M2 agonist treatment. The analysis of cell migration and morphology allowed us to demonstrate that M2 receptor activation caused an arrest of SC migration and modified cell morphology probably by the modulation of β1-arrestin/cofilin-1 and PKCα expression, respectively. Conclusions: The data obtained demonstrated that M2 receptor activation in addition to the canonical Gi protein-coupled pathway modulates noncanonical pathways involving the mTORC1 complex and other kinases whose activation may contribute to the inhibition of SC proliferation and migration and address SC differentiation.

## 1. Introduction

In the peripheral nervous system (PNS), Schwann cells (SCs) play an essential role both during development and regeneration of the peripheral nerves. Schwann cells are responsible for the formation of the myelin sheaths that envelope the axons, making the transmission of nerve impulses more efficient. These glial cells are derived from the embryonic neural crest and then differentiate into myelinating and non-myelinating Schwann cells [1]. SCs have remarkable plasticity; in fact, in response to damage, injury or genetic manipulation, Schwann cells de-differentiate and acquire new molecular and functional characteristics in order to coordinate the response to damage, through processes of myelinophagy, secretion of cytokines and neurotrophic factors supporting axonal regeneration [2,3,4]. Previous studies demonstrated how different neurotransmitters can control and modulate biological processes, both during PNS development and in adult life [5,6,7]. It has been shown that both rat and human SCs express muscarinic and nicotinic receptors and are able to respond to ACh stimuli [8,9,10,11]. Among the muscarinic receptor subtypes, the most abundant is the M2 subtype [8]. M2 mAChRs belong to the superfamily of G protein-coupled receptors (GPCRs). Canonically, once activated, the receptor transduces a signal downstream by interacting with the inhibitory GTP-binding protein, G_i_. This G_i_ protein inactivates the enzyme adenylate cyclase, leading to a decrease in intracellular cAMP levels, known to be important for cell proliferation in SCs [12,13]. Once the receptor has been activated, the desensitization process begins; the phosphorylation of M2 mAChR leads to recruitment of the β-arrestin proteins, which causes dissociation of the receptor from the G_i_ protein, promoting internalization of the receptor and consequently its desensitization [14,15,16]. Furthermore, several authors suggest that β-arrestins not only promote the internalization of activated GPCRs, but also may act as scaffolds for kinases such as c-Jun N-terminal kinases (JNK1, JNK2, JNK3), Raf, ERK 1/2 and PI3K, AKT, leading to the activation of signaling pathways independently of G protein activity [17,18].

Our previous studies have demonstrated that the activation of the M2 receptor in SCs with the orthosteric agonist arecaidine propargyl ester (APE) led to a decrease in cAMP levels without intracellular calcium spikes, according to the canonical signaling mediated by Gi-proteins [8,9,10,11]. In addition, the activation of M2 mAChR using the selective agonist APE induced reversible cell cycle arrest in the G1-S phase and caused a slowing proliferation rate, promoting rat SCs differentiation towards the myelinating phenotype, with the upregulation of promyelinating transcription factors such as Sox10 and Egr2, followed by increased expression of myelin proteins [8,9,10]. Considering that M2 mAChRs negatively modulate proliferation and promote differentiation of rat SCs, the aim of this work was to better characterize the transduction pathways activated downstream M2 receptor activation through M2 agonist APE. The effects of M2 receptor activation on the PI3K/Akt/mTOR pathway known to be involved in the control of the SC proliferation and myelination were evaluated [19,20]. Moreover, the possible relationship between signaling pathways M2 receptor-activated and the modulation of SC migration and cell morphology changes were also investigated.

## 2. Materials and Methods

### 2.1. Statements for Animal Use

The procedures involving animals were carried out in accordance with the guidelines of the European Communities Council Directive (86/609/EEC of 24 November 1986) and Italian National Law DL/116/92. All methods were in accordance with guidelines of the protocol n. 7FF2C.6.EXT.96 that was approved by the Ministry of Health (Aut. N. 1184/2016-PR 16/12/2016). All animals were housed in a temperature-controlled room (22 ± 1 °C) with a 12 h light/dark cycle and free access to food and water.

### 2.2. Cell Cultures

Primary Schwann cells were isolated from sciatic nerves of 2-days-old Wistar pups, as previously described [8]. Briefly, sciatic nerves were removed and collected in fresh high-glucose Dulbecco’s Modified Eagle’s Medium (DMEM, Sigma-Aldrich) with Hepes 25 mM. Subsequently, sciatic nerves were digested with trypsin/collagenase (Type I, Sigma-Aldrich, St. Louis, MA, USA) and seeded into T25 flasks with fresh DMEM containing 10% fetal bovine serum (FBS, Immunological Sciences, Milan, Italy). To selectively remove fibroblasts, cells were treated with 1 mM cytosine arabinoside (AraC, Sigma-Aldrich, Milan, Italy) for 48 h and then with anti-Thy 1.1 (1:1000, Serotec, Bio-Radgroup, Hercules, CA, USA) and rabbit complement (1:2 *v*/*v*) (Cedarlane, Burlington, ON, Canada). SCs were then amplified in DMEM, 10% FBS, 5 μM forskolin (Fsk; Sigma-Aldrich, Milan, Italy) and bovine pituitary extract (1:150, Sigma-Aldrich). SCs were incubated in 10% ${\mathrm{CO}}_{2}$ at 37 °C and maintained at sub-confluent levels onto Poly-L-Lysine-coated 75 ${\mathrm{cm}}^{2\;}$ flasks (Sigma-Aldrich, Milan, Italy). SCs were amplified in DMEM without sodium pyruvate, 10% FBS supplemented with 1% streptomycin, 50 IU/mL penicillin (Sigma-Aldrich, Milan, Italy), 1% glutamine (Sigma-Aldrich), 2 μM forskolin and 10 ng/mL Neuregulin-1 (Immunological Sciences, Milan, Italy).

### 2.3. Pharmacological Treatment

SCs were treated with the preferential agonist of M2 receptor subtype, Arecaidine Propargyl Ester hydrobromide (APE, Sigma-Aldrich, Milan, Italy), at the final concentration of 100 μM. The concentration was established by previous studies demonstrating that 100 μM of APE was able to produce the best effects in terms of inhibition of cell proliferation and upregulation of differentiation factors expression. Moreover, pharmacological binding experiments and M2 knockdown with short interference RNA technique demonstrated that APE selectively binds M2 muscarinic subtype in SCs as well as in other cell models [8,9,10,11,21,22,23,24,25].

For the concentrations of the muscarinic antagonists, we referenced previous studies of competition binding experiments that have allowed us to identify a value close to the IC50 of displacement of the muscarinic agonist QNB [26,27]. The muscarinic antagonists and their respective concentrations used were: Pirenzepine (M1 antagonist) final concentration 0.1 μM, Methoctramine (M2 antagonist) final concentration 0.1 μM, 4-DAMP (M3 antagonist) final concentration 0.01 μM, Muscarine (Sigma-Aldrich, Milan, Italy), nonselective muscarinic receptor agonist, final concentration 100 μM. Chelerythrine Chloride (1 μM) (Sigma-Aldrich, Milan, Italy) was used as a PKCα inhibitor [28]. Controls were obtained maintaining the cells in only growth medium. All experiments were performed in triplicate.

### 2.4. Western Blot Analysis

Cells were homogenized with a lysis buffer (Tris-EDTA 10 mM, 0.5% NP40, NaCl 150 mM) containing a protease inhibitor cocktail (Sigma-Aldrich, Milan, Italy). Lysates were incubated for 20 min on ice, sonicated for 15 s and then centrifuged for 10 min at 14,000 rpm at 4 °C. Protein concentration was determined using a BCA Protein Assay Kit (Thermo Scientific, Waltham, MA, USA), according to the manufacturer’s protocol. A sample buffer supplemented with 5% β-mercaptoethanol was added to protein lysates and heated for 5 min at 95 °C, loaded onto an 8 % on SDS-polyacrylamide gel (SDS-PAGE) and run at 100 V using a running buffer (0.25 M Tris, 2.4 M Glycine, 0.035 M SDS). SDS-PAGE gels were transferred to Polyvinylidene Difluoride (PVDF) sheets (Merck Millipore, Darmstadt, Germany) at 80 V in a transfer buffer (20 mM Tris; 150 mM glycine, 5% [*v/v*] methanol) for 60 min at 4 °C. Membranes were blocked for 60 min in 5% of non-fat milk powder (Sigma-Aldrich, St. Louis, MO, USA) in PBS containing 0.1% Tween-20 and then incubated at 4 °C overnight, with the antibodies previously diluted in the blocking solution. Primary antibodies used were anti-Arrestin Beta 1 antibody (dilution 1:2000, Immunological Science, RM, Italy, AB-84290), anti-PI3 Kinase p85 (19H8) (dilution 1:800, Cell Signaling Technology, MA, USA, #4257), anti-Phospho-AMPKα (thr172) (40H9), (dilution 1:800, Cell Signaling Technology, MA, USA, #2535), anti-Phospho-Akt (thr308) (244F9), (dilution 1:800, Cell Signaling Technology, MA, USA, #4056), anti-Phospho-Akt (Ser473) (193H12), (dilution 1:800, Cell Signaling Technology, MA, USA, #4058), anti-Akt (pan) (11E7), (dilution 1:800, Cell Signaling Technology, MA, USA, #4685), anti-Phospho-p-70 S6 Kinase-T389 (dilution 1:600, Immunological Science, Milan, Italy, MAB-94648), anti-PRKCA (dilution 1:2000, Immunological Science, Milan, Italy, AB-84289), anti-α-cofilin 1 (dilution 1:1000, Merk Millipore, MA, USA, AB3842). β-Actin (Immunological Sciences, Milan, Italy) was used as the reference protein. After overnight incubation, membranes were washed three times with PBS + 0.1% Tween-20 buffer and then incubated for 1 h at room temperature (RT) with secondary antibodies conjugated to horseradish peroxidase/anti-rabbit horseradish peroxidase (1:10,000, Promega, Milan, Italy) or anti-mouse horseradish peroxidase (1:10,000, Immunological Sciences, Milan, Italy). Membranes were exposed to ECL chemiluminescence reagent (Immunological Sciences, Milan, Italy) for signal detection. The intensity of the bands was evaluated by exposure to Chemidoc (Molecular Imager ChemiDoc XRS + System with Image Lab Software, Bio-Rad, CA, USA). Densitometry analyses were performed using ImageJ imaging software (NIH, Bethesda, MD, USA).

### 2.5. Immunocytochemistry

Schwann cells were plated on Poly-l-Lysine-coated coverslips arranged in 24-well plates at the density of 2 × $10^{4}$ cells in DMEM containing 10% FBS, 2 μM forskolin and 10 ng/mL Neuregulin-1. Cells were maintained for 48 h either in the presence or absence of 100 μM APE. Cells were washed three times with PBS and fixed with 4% paraformaldehyde in PBS for 20 min at RT. After three washes in PBS, cells were pre-incubated in PBS solution containing 0.1% Triton X-100, 1% bovine albumin serum (BSA) and 10% normal goat serum (NGS) for 60 min at RT. Cells then were incubated with the primary antibodies: anti-GFAP (1:400, Immunological Sciences, Milan, Italy, AB-10682), anti-Tubulin alpha (1:40, Immunological Sciences, Milan, Italy, MAB-94264) at 4 °C overnight, diluted in 1% NGS and 1% BSA in PBS. After overnight incubation, cells were washed three times with PBS and incubated with Alexa-488-conjugated goat anti-rabbit (IgG diluted 1:200 in PBS + 0.1% Triton X-100 + 1% NGS; Immunological Sciences) and Alexa-594-conjugated goat anti-mouse (diluted 1:200 in PBS + 0.1% Triton X-100 + 1% NGS; Immunological Sciences) for 2 h at RT. After three washes in PBS, the cells were mounted with Anti-Fade Mounting Medium with 4′,6-diamidino-2-phenylindole (DAPI, Immunological Science). The images were acquired with a Zeiss Apotome fluorescence microscope, using a 63× objective through the Axion Vision program (Zeiss, Oberkochen, Germany).

### 2.6. Wound Healing

Cell migration was evaluated through a wound healing assay. Cells were plated on poly-l-Lysine-coated dishes (35 mm diameter) at the density of 3 × $10^{5}$ cells in complete growth medium. The treatments were added two hours before the scratches were performed; the antagonists were added 1 h before the agonist treatment. After two hours of treatment, a scratch was generated with a p200 tip and, to exclude possible proliferation interference, Mitomycin C was also added to the cell culture media (50 ng/mL, Sigma-Aldrich, Milan, Italy). The cells were photographed at time 0 h and after 6 h from the scratch using a 10× magnification objective. Images were obtained using an optic microscope (Zeiss, Oberkochen, Germany). For each well, three pictures were taken (up, middle and the bottom of the scratch line). The space between two fronts at time 0 (t0) and after 6 h (t6) was measured in all experimental conditions using ImageJ imaging software (NIH, Bethesda, MD, USA). Three measurements (up, middle and the bottom of the scratch line) were obtained for each picture. Each treatment was performed in technical triplicate and then in experimental triplicate. To measure the width of the scratch, a line was drawn between the two cell fronts. The average t0 value for each treatment was subtracted to the t6 measurements of the referred treatment, obtaining the measure of the covered space by the cells.

### 2.7. Statistical Analysis

Data analyses were performed with GraphPad Prism 8.0.1 (GraphPad Software Inc, La Jolla, CA, USA). Data are presented as the average ± SEM. Student’s *t*-test or one-way ANOVA analyses were used to evaluate statistical significance within the different samples. A value of *p* < 0.05 was considered statistically significant; *p* < 0.05 (*); *p* < 0.01 (**); *p* < 0.001 (***).

## 3. Results

### 3.1. Analysis of Signaling Pathways Downstream M2 Receptor Activation

**Figure 1:**
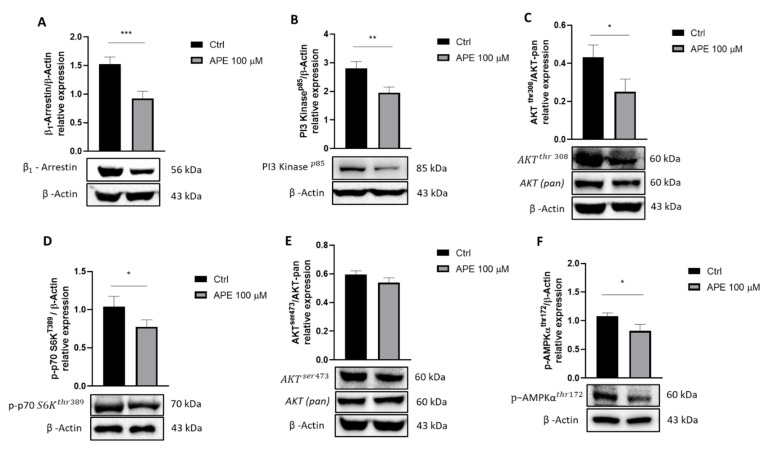
Western blot analysis of several signal transduction effectors upon 48 h of 100 μM APE treatment. (**A**) $\beta_{1}$-arrestin expression. β-Actin was used as reference protein. The graph shows the densitometric analysis of the bands normalized with the reference protein β-Actin. (**B**) ${\mathrm{PI}3\;\mathrm{Kinase}\;}^{p85}$ expression. β-Actin was used as reference protein. The graph shows the densitometric analysis of the bands normalized with the bands of the reference protein β-Actin. (**C**) $AKT^{Thr\;308}$ expression. AKT (pan) and β-Actin were used as reference protein. The graph shows the densitometric analysis of the bands normalized with the bands of the reference protein AKT (pan). (**D**) $\mathrm{p-p}70\;S6K^{Thr389}$ expression. β-Actin was used as reference protein. The graph shows the densitometric analysis of the bands normalized with the bands of the reference protein β-Actin. (**E**) $AKT^{ser473}$ expression. AKT (pan) and β-Actin were used as reference proteins. The graph shows the densitometric analysis of the bands of Western blot analysis for $AKT^{ser473}$ normalized with the bands of the reference protein AKT (pan). (**F**) $\mathrm{p-}{\mathrm{AMPK}\alpha }^{thr172}$ expression. β-Actin was used as reference protein. The graph shows the densitometric analysis of the bands of Western blot analysis for $\mathrm{p-}{\mathrm{AMPK}\alpha }^{thr172}$ normalized with the bands of the reference protein β-Actin. All the data are the average ± SEM of three independent experiments. Student’s *t*-test was used (* *p* < 0.05; ** *p* < 0.01; *** *p* < 0.001).

In addition to the canonical signaling pathway, the M2 receptor, as the other GPCRs, can activate G protein-independent signaling pathways or involve multifunctional adaptor proteins as β-arrestins. In fact, recent studies have proposed that activated GPCR could lead to the formation of a “supercomplex” where GPCR and $\beta_{1}$-arrestin give rise to a unique signaling pathway together with that derived from G protein activation [29,30]. Starting from this knowledge, we analyzed a possible modulation of proteins downstream of the $\beta_{1}$-arrestin pathway following treatment with M2 orthosteric agonist APE, investigating a possible modulation of the PI3K/Akt/mTORC1 signaling pathway. By Western blot analysis, we evaluated $\beta_{1}$-arrestin protein expression. As shown in Figure 1A, $\beta_{1}$-arrestin expression decreased significantly after 48 h of APE treatment. β-arrestins can regulate PI3K activity both positively and negatively depending on the activating receptor. Based on these results, the expression of ${\mathrm{PI}3\;\mathrm{Kinase}\;}^{p85}$ was analyzed. As shown in Figure 1B, APE treatment caused downregulated expression of ${\mathrm{PI}3\;\mathrm{Kinase}\;}^{p85}$. The same trend was observed for the expression of Phospho-Akt protein (Thr308 phosphorylated) compared to that of Akt (pan), which did not discriminate between the active and inactive form (Figure 1C). To elucidate the involvement of the PI3K/Akt/mTORC1 pathway in this signaling, we analyzed the expression of the mTORC1 downstream substrate, $\mathrm{p-p}70\;S6K^{Thr389}$. Western blot analyses showed decreasing levels of phosphorylation of this substrate (Figure 1D).

The mTORC2 complex is able to phosphorylate the kinase Akt at Ser 473 [31], so in order to investigate whether M2 receptor activation also causes possible modulation of the mTORC2 complex, we analyzed the expression of Akt phosphorylated at Ser 473 compared to that of Akt (pan). Western blot analysis showed no significant differences between APE-treated and untreated cells (Figure 1E).

As described above, activation of the M2 receptor results in an increase of myelin-constituting proteins such as P0, PMP22 and MBP and of differentiation-promoting factors such as Sox10 and Krox20. This may suggest that the activation of the M2 receptor could promote a differentiating program [10]. To confirm these data, we analyzed the expression of AMP-activated protein kinase (AMPK) phosphorylated at Thr 172. It is well-known that AMPK in sciatic nerves gradually decreases during the PNS myelination process, suggesting that this kinase is an important negative regulator of myelination [32]. As shown in Figure 1F, APE treatment caused downregulated expression of $\mathrm{p-}{\mathrm{AMPK}\alpha }^{thr172}$, confirming the involvement of the M2 receptor in the SC differentiation program.

### 3.2. M2 Receptor Activation Results in SC Migration Arrest

**Figure 2:**
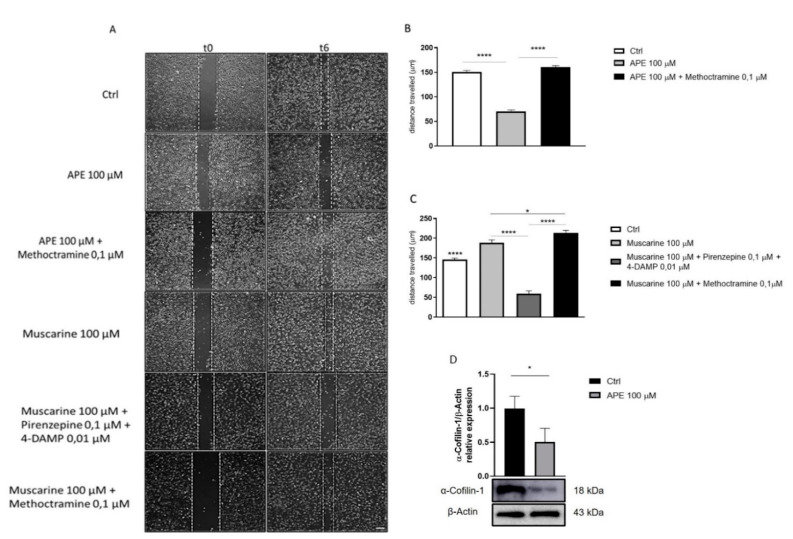
Analysis of SC migration. (**A**) The distance of the gap between two fronts was measured at two different time points (t0–t6); (scale bar = 100 µm); (**B**) M2 activation by the selective agonist APE 100 μM was able to reduce SC migration; this effect was counteracted by the M2 antagonist methoctramine 0.1 μM (**** *p* < 0.0001); (**C**) treatment with Muscarine 100 μM, a nonselective muscarinic receptor agonist, promoted cell migration compared to the control (**** *p* < 0.0001 Ctrl vs. Muscaine); treatment with Muscarine 100 μM + Pirenzepine 0.1 μM (M1 muscarinic receptor antagonist) + 4-DAMP 0.01 μM (M3 muscarinic receptor antagonist) caused a decrease in cell migration, (**** *p* < 0.0001 Muscarine 100 μM vs. Muscarine 100 μM + Pirenzepine 0.1 μM +4-DAMP 0.01 μM); treatment with Muscarine 100 μM + Methoctramine 0.1 μM (M2 muscarinic receptor antagonist) was able to promote cell migration, inhibiting the M2 effect, (* *p* < 0.05 Muscarine 100 μM vs. Muscarine 100 μM + Methoctramine 0.1 μM). The data obtained are the average ± SEM of at least three independent experiments carried out in triplicate. (**D**) α-cofilin-1 expression by Western blot analysis in Schwann cells after 48 h of 100 μM APE treatment. β-Actin was used as the internal reference protein. The graph shows the densitometric analysis of the bands of Western blot analysis for α-cofilin-1 normalized with β-Actin. The data are the average ± SEM of three independent experiments. Student’s *t*-test was used * *p* < 0.05.

The activation of the PI3K/Akt pathway also induces activation of p70 S6K, causing actin remodeling and promoting cell migration and invasion [33]. Because M2 receptor activation causes a decrease in PI3K/AKT/$\mathrm{p-p}70\;S6K^{Thr389}$ protein levels, to determine whether the selective activation of the M2 receptor would influence cell migration, a wound healing assay was performed. SCs were analyzed 6 h after the scratch. As shown in Figure 2A, APE treatment negatively modulated cell migration. Migration was also measured in the presence of the M2 antagonist methoctramine (0.1 μM); the M2 antagonist was able to counteract the APE effect and, in this condition, the migration was comparable to that of the untreated cells (Figure 2B). Cell migration was further assessed by treatment with muscarine 100 μM alone, a nonselective muscarinic receptor agonist (Figure 2A), or by co-treatment with muscarine 100 μM and M1 muscarinic receptor antagonist Pirenzepine (0.1 μM) and the M3 muscarinic receptor antagonist 4-DAMP (0.01 μM) (Figure 2A,C). Furthermore, co-treatment with muscarine 100 μM and the M2 receptor antagonist methoctramine (0.1 μM) (Figure 2C) was performed. Antagonists were added to the culture medium 1 h before the treatment with agonists. As shown in Figure 2C, muscarine treatment, by activating all muscarinic receptors, induced an increase in cell migration compared with that of control. The antagonists Pirenzepine and 4-DAMP resulted in reduced cell migration, suggesting the involvement of the M1 and M3 muscarinic receptor subtypes in promoting cell migration (Figure 2C). Otherwise, co-treatment with muscarine and the M2 antagonist methoctramine promoted cell migration (Figure 2C). As shown above, activation of the M2 receptor by the APE agonist caused a decrease in $\beta_{1}$-arrestin protein levels. β-arrestins play a critical role in cell migration downstream of multiple G protein-coupled receptors (GPCRs) through multiple mechanisms, such as the regulation of actin assembly by interacting with cofilin [34]. To investigate whether $\beta_{1}$-arrestin modulation by M2 agonist APE may influence α-cofilin-1 expression, we performed a Western blot analysis of unphosphorylated α-cofilin-1 levels. The result showed a significant reduction in unphosphorylated α-cofilin-1 levels in SCs after APE treatment compared to the control (Figure 2D).

### 3.3. M2 Receptor Activation Induces Morphological Change in SCs

**Figure 3:**
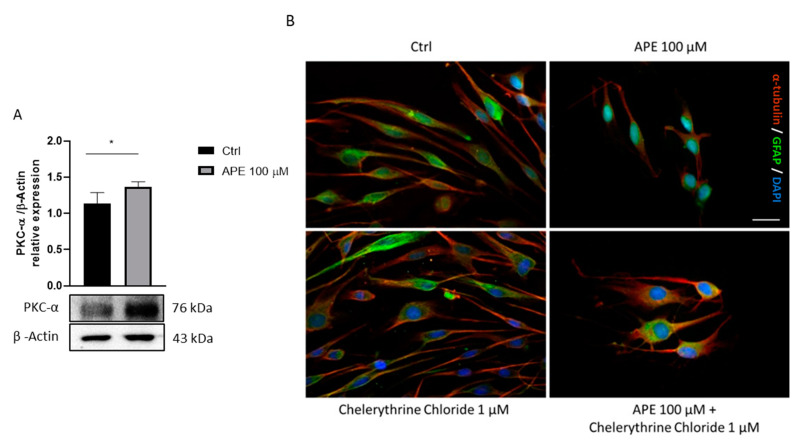
(**A**) PKCα kinase expression by Western blot analysis in Schwann cells after 48 h of 100 μM APE treatment. β-Actin was used as reference protein. The graph shows the densitometric analysis of the bands of Western blot analysis for PKCα kinase normalized with the bands of β-Actin used as the reference protein. The data are the average ± SEM of three independent experiments. Student’s *t*-test was used, * *p* < 0.05. (**B**) Immunocytochemistry for α-tubulin and GFAP in control condition (10% FBS + 2 μM fsk and 10 ng/mL Neuregulin-1), after 100 μM APE; 1 μM Chelerythrine Chloride; 100 μM APE plus 1 μM Chelerythrine Chloride (scale bar = 20 μm). SCs in control condition showed a bipolar elongated morphology, compatible with the immature phenotype; SCs after APE 100 μM showed a rounded morphology; SCs after co-treatment with APE 100 μM and Chelerythrine Chloride 1 μM showed an intermediate morphology between ctrl and APE treatment.

Previous studies showed that the activation of the M2 receptor leads to cytoskeletal modifications, with changes in cell morphology and redistribution of adhesion molecules [10]. It is known that PKC-α sits at the crossroads of many signal transduction pathways and is implicated in a wide range of cellular responses. Various PKC isoforms drive a glycogenesis program associated with cytoskeletal remodeling, cell cycle control and metabolism modifications [35,36]. As shown in Figure 3A, APE treatment caused a significant increase in PKCα kinase protein levels compared to the untreated cells. To determine whether the activation of the M2 receptor involves the PKCα in cytoskeletal remodeling, immunocytochemical analyses were performed after 48 h of treatment with the selective agonist APE 100 μM and with the PKCα inhibitor Chelerythrine Chloride 1 μM (Figure 3B). Staining with α-tubulin and GFAP in untreated SCs showed a classic bipolar elongated morphology. When SCs were cultured in the presence of APE 100 μM for 48 h, they appeared as flat cells, progressively losing the classic bipolar morphology. However, staining with α-tubulin and GFAP following treatment with the PKCα inhibitor Chelerythrine Chloride 1 μM showed a cell morphology comparable to the untreated cells. Co-treatment with APE and Chelerythrine Chloride reduced the morphological changes induced by APE treatment. This result suggests that the remodeling of the cytoskeleton caused by the activation of the M2 receptor may involve PKCα, in addition to other possible effectors (Figure 3B).

## 4. Discussion

In the past few years, it has been shown that various neurotransmitters are involved in SC development and differentiation [5]. Among these, acetylcholine plays a crucial role. Activation of the M3 and M2 receptors has been shown to regulate proliferation in both oligodendrocytes (OLs) and SCs, respectively [8,9,10,11,37]. Specifically, the M2 receptor activation, the most abundant muscarinic receptor subtype expressed in SCs, caused reversible arrest of cell cycle progression, with accumulation of SCs in the G1 phase, and additionally promoted the expression of transcription factors involved in SCs differentiation, such as Sox10 and Krox20/egr-2 [8,9]. Krox-20 is considered to be the main regulator of myelin, promoting cell cycle exit and triggering the myelination transcription factors Oct-6 and Brn-2 [38,39]. Oct-6 is only required for the initial phases of myelin signaling, with Krox20 and Sox10 involved in the formation and maintenance of the myelin [40,41,42]. The development of SCs, from immature to myelinating cells, requires a multitude of regulatory signals [43]. Since M2 muscarinic receptors contribute to regulating SC differentiation and myelin organization [10], we analyzed one of the pathways most involved in myelination, the PI3K/Akt/mTORC1 pathway [19,20,43,44,45]. This pathway is known to be directly involved in the regulation of myelination in Schwann cells, especially in the early stage [20,46]. This signaling is required for the proliferation of Schwann cells in vitro [20], and its inhibition is necessary to initiate myelination but is not required in myelin maintenance. Recent studies have shown that mTORC1 activity is tightly associated with the expression of the transcription factor Krox-20 through S6K [19]. Indeed, it has been shown that high mTORC1 activity suppresses the expression of Krox20, the master transcription factor of myelinating SCs, thus inhibiting SC differentiation and the initiation of the myelination process. In fact, before the onset of myelination, high levels of mTORC1 activity coincide with the period of intense radial sorting to ensure that myelination was activated only after radial sorting was completed [19]. On the contrary, low levels of mTORC1 allow the expression of the transcription factor Krox-20, allowing SC differentiation and thus the initiation of myelination. Based on this dual role of the PI3K/Akt/mTORC1 axis, we evaluated a possible involvement of M2 receptor activity in the modulation of this pathway.

GPCRs can interact with a variety of different proteins in addition to heterotrimeric G proteins, thus allowing different responses other than canonical signaling pathways [47,48,49]. Starting from this knowledge, in addition to the canonical pathway promoted by M2 receptor activation, which, blocking adenylate cyclase, leads to a decrease of cyclic AMP (cAMP) levels through coupling with Gi-proteins, [9], we assessed the activation of an alternative G protein-independent pathway promoted by the M2 receptor. One of these involved multifunctional adaptor proteins β-arrestins. These proteins bind the phosphorylated receptor, blocking the receptor through a steric mechanism. Following the attack of the β-arrestins at the receptor, this may recruit adaptor proteins (AP2) and clathrins for the internalization and desensitization of the receptor [17,18,47,50]. In recent years, a further role of β-arrestins as a signal transduction scaffold was reported; in fact β-arrestins can act as scaffold for ERK1/2, c-Jun N- terminal kinases and PI3K/AKT pathways [51]. Recent studies have highlighted the crucial role of the PI3K/Akt/mTORC1 pathway in regulating PNS myelination [19,20,44,45]. Phosphatidylinositol 3-kinases (PI3K) activation allows phosphoinositide-dependent protein kinase-1 (PDK1) to activate Akt by phosphorylation at Thr308 [52].

These latest findings led us to investigate possible effects of the M2 receptor activation in altering $\beta_{1}$-arrestin and PI3K/AKT pathways. Given the high degree of structural similarity between the different muscarinic receptor subtypes (M1, M2, M3, M4 and M5), it is difficult to find selective agonists and antagonists for each receptor type [13]. Previously, our group extensively investigated the effects of the preferential M2 agonist, arecaidine propargyl ester (APE) in rat and human SCs. Pharmacological binding experiments and M2 receptor knockdown showed that APE, a synthetic arecaidine derivate, is selective for the M2 receptor also when used at the high dose of 100 µM [9,21,22]. These results prompted us to use APE as a preferred M2 receptor subtype agonist also in this study. Our results showed a significant decrease in $\beta_{1}$-arrestin protein expression upon M2 agonist treatment. Similarly, it was observed that PI3K and $AKT^{thr308}$, its specific targets, were significantly reduced. This finding may suggest the consequential decrease of ${\mathrm{PI}3\;\mathrm{Kinase}\;}^{p85}$ and $AKT^{thr308}\;$protein levels, downstream M2 receptor activation and $\beta_{1}$-arrestin downregulation.

In the literature, it is known that the PI3K protein is crucial in directing the Akt/mTORC1 pathway during proliferation and then SC differentiation. In particular, the inhibition of the PI3K protein is required at the beginning of myelination to downregulate the Akt/mTORC1 pathway [43,53]. The ability of the M2 receptor to downregulate this pathway is in accordance with our previous observation indicating the ability of this muscarinic receptor subtype to modulate SC differentiation [10].

To better elucidate the role of mTORC1 signaling downstream to the muscarinic receptor, we evaluated, upon APE treatment, the phosphorylation of $\mathrm{p-p}70\;S6K^{Thr389}$, a specific downstream substrate of mTORC1. Our analysis showed a significant decrease of $\mathrm{p-p}70\;S6K^{Thr389}$ protein levels. This result correlates with findings in the literature indicating that the activity of mTORC1 is progressively reduced during SC differentiation in order to remove its inhibition on Krox-20/erg2 expression [54]. M2 receptors cause the increase of Krox20/egr2 expression [10]. This suggests that the reduced mTORC1 levels present upon M2 receptor activation may drive the myelination process [19,43].

The mTOR kinase consists of two multi-protein complexes, mTORC1 and mTORC2; these have different regulation, downstream targets and sensitivity to the allosteric mTOR inhibitor rapamycin [54]. Firstly, we evaluated the involvement of mTORC2, analyzing the protein levels of its downstream target $AKT^{ser473}\;$following treatment with the M2 receptor agonist, APE. In this case, no significant difference was observed after the M2 agonist treatment, indicating no direct involvement of mTORC2 in these events. It is reported that the absence of mTORC2 signaling does not interfere with SC differentiation or myelination [43].

AMP-activated kinase (AMPK) is a heterotrimeric serine/threonine protein kinase that could be stimulated by nutritional status such as a low ratio of ATP to AMP, promoting catabolic processes to produce energy. AMPK can act as a negative regulator of myelination, inhibiting lipid and protein synthesis and, most importantly, myelin genes expression through c-Jun upregulation [32,55]. Our results have shown a decrease in $\mathrm{p-}{\mathrm{AMPK}\alpha }^{thr172}$ protein levels following treatment with M2 agonist APE, according to the decrease of c-jun expression after M2 receptor activation [10].

All these results demonstrate the ability of the M2 agonist to activate complex signaling transduction pathways in SCs, strongly supporting the pro-differentiating effects modulated by the M2 receptor [10].

The SC differentiation process consists of three main phases, proliferation, pro-myelination and myelination [56,57,58]. The proliferative phase is characterized by cell division and migration of Schwann cells, whereas the pro-myelinating and myelinating phases are characterized by dynamic SC morphological changes. Starting from our previous studies demonstrating the reversible arrest of SC proliferation upon M2 agonist treatment [9], here we studied the other two fundamental aspects related to the differentiation process: cell migration and morphological changes. Our results showed that the activation of the M2 muscarinic receptor induced an arrest of SC migration. In order to confirm that the effects are mediated by M2 receptors, we evaluated the ability of different muscarinic antagonists to counteract the APE-induced effects. Albeit the pharmacological antagonists for the muscarinic receptors presented poor selectivity, the competition binding experiments and the value of the IC50 obtained in our previous studies performed in different cell models (i.e., sensory neurons, glioblastoma and neuroblastoma cells) have indicated the value that may be considered selective for the specific receptor subtypes [26,27]. Using the different muscarinic antagonists to the concentrations mentioned in the methods, we observed that only methoctramine, an M2 receptor antagonist, counteracted the APE effects. In addition, this effect was further confirmed by common activation of all muscarinic receptor subtypes by treatment with the nonselective agonist muscarine, alone and in the presence of antagonists of the M1 and M3 receptors, pirenzepine and 4-DAMP, respectively, and in the presence of an M2 receptor antagonist methoctramine. These experiments confirmed the direct involvement of the M2 receptor in the inhibition of SC migration; instead, the M1 and M3 receptor subtypes may play an opposite role. PI3K is required for cell migration because it can contribute to actin filament remodeling through Akt and p70S6K1 [59,60,61]. Additionally, β-arrestins play a critical role in cell migration downstream of multiple G protein-coupled receptors (GPCRs) through multiple mechanisms [34]. In this way, downregulation of $\beta_{1}$-arrestin expression and the possible consequent downregulation of the PI3K/Akt pathways downstream M2 receptor activation could in part explain the decreased cell proliferation previously demonstrated [8,9] and the arrest of SC migration. Many studies indicate that β-arrestins have an important role in actin assembly. This event is the main driving force of cellular movement promoting stress fiber, lamellipodia and filopodia formation [62]. β-arrestins are capable of interacting with cofilin, facilitating its dephosphorylation with its upstream phosphatases (i.e., Chronophin and Slingshot) and thus promoting its activation [62,63]. Our results showed a decrease in the expression of non-phosphorylated α-cofilin-1, suggesting a possible reduction of its activity β1-arrestin mediated that may further support the arrest of SC migration.

It is known that various PKC isoforms drive a glycogenesis program mediating cytoskeletal remodeling, cell cycle control and metabolism modifications [35,36]. APE treatment leads to cytoskeletal modifications, with changes in cell morphology and redistribution of adhesion molecules [10]. In a possible framework of cell differentiation, we analyzed the protein expression of the PKCα following treatment with the selective agonist, APE. After 48 h of treatment, the PKCα protein levels were upregulated in the treated cells compared to the control. Based on these data, we assessed whether increased PKCα protein expression following treatment with the selective agonist APE was correlated with morphological changes. To test this hypothesis, SCs were treated with the PKCα inhibitor, Chelerythrine Chloride (CC), and then a morphological analysis was performed. The morphological changes observed in SCs treated with the M2 agonist, APE, were partly reversed by treatment with the PKCα inhibitor, confirming a possible participation of this kinase in cytoskeletal remodeling following M2 receptor activation. In fact, albeit CC was described as a specific inhibitor of PKCα [28,64,65], it is not possible to exclude a participation of other isoforms of this enzyme in regulating the SC morphological changes that theM2 receptor mediated.

## 5. Conclusions

**Figure 4:**
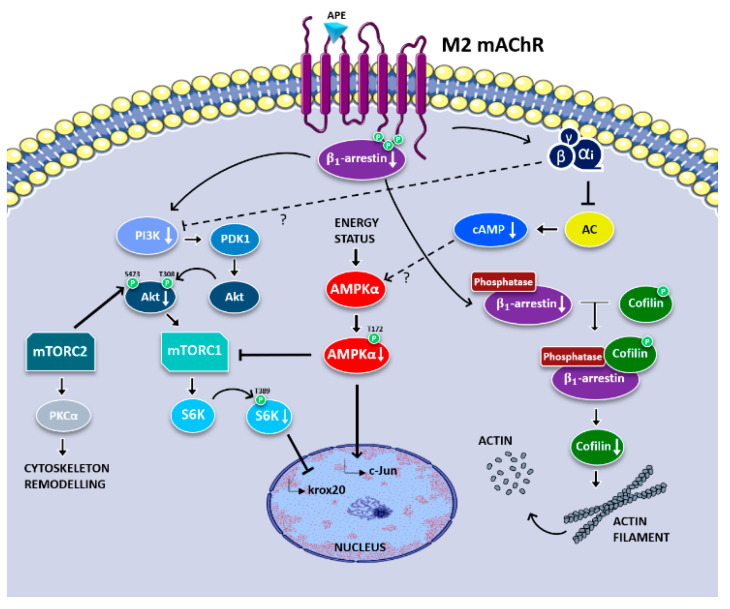
Schematic representation of signaling pathways downstream the M2 muscarinic receptor activation modulating SC proliferation/differentiation. In addition to the canonical pathway mediated by Gαi, M2 receptors may modulate the PI3K/Akt pathway via β1-arrestin or/and βγ-subunits of Gi-proteins, causing downregulation of mTORC1 pathway activity. This complex regulation mediated by M2 receptor activation may explain the decreased SC proliferation and the upregulation of the differentiation factors. β1-arrestin downregulation may also contribute to the modulation of the SC morphological changes, influencing the cofilin phosphorylation and actin de-polymerization with possible consequence on SC migration.

The data reported in the present work demonstrated the ability of M2 receptors to activate canonical and noncanonical signaling transduction pathways involved in the regulation of different phases of SC differentiation. In fact, M2 receptor activation, activating the canonical Gi-protein-coupled pathway, is responsible for negatively modulating cAMP levels [8]. It can also negatively modulate the PI3K/Akt/mTORC1 axis, possibly through a noncanonical pathway mediated by $\beta_{1}$-arrestin (Figure 4). The downregulation of this pathway contributes to promoting the myelinating SC phenotype. These results are also confirmed by the downregulated expression of $\mathrm{p-}{\mathrm{AMPK}\alpha }^{thr172}$, a negative regulator of myelination, by the negative modulation of cell migration and by changes of SC morphology. Altogether, these data support and explain the molecular mechanisms downstream M2 receptor activation able to control the SC switching from the immature-proliferating phase to the myelinating phenotype previously described by our group [10]. However, it is not possible to exclude the participation of other signal transduction pathways. Further investigations will be necessary to address the possible involvement of other kinases or of small G proteins of the Rho family.
